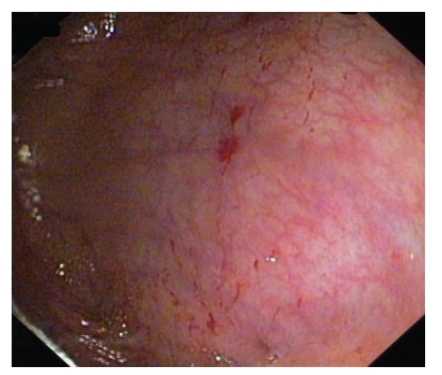# Cat Scratch Colon

**DOI:** 10.1155/2011/875941

**Published:** 2011-04-28

**Authors:** M. Lourdes Ruiz-Rebollo, Benito Velayos-Jiménez, José María Prieto de Paula, María Álvarez Quiñones, José Manuel González Hernández

**Affiliations:** ^1^Hepatogastroenterology Department, Clinical University Hospital, Valladolid, Spain; ^2^Internal Medicine Department, Clinical University Hospital, Valladolid, Spain; ^3^Pathology Department, Clinical University Hospital, Valladolid, Spain

## Abstract

Over the past few years, we have read several publications regarding the term “cat scratch colon.” This neologism was developed to define some bright red linear markings seen in the colonic mucosa that resemble scratches made by a cat. We would like to communicate a recent case attended at our institution.

## 

A 67-year-old man with abdominal pain was referred to our unit for colonoscopy. He was a heavy smoker and a moderate drinker. He suffered from peripheral vascular disease and was treated with pentoxifylline and acetyl salicylic acid. He had been operated on his stomach due to a gastric peptic perforation years ago; he had a pulmonary benign tumour resected and a hepatic hydatid cyst removed several years ago. No abnormalities were seen in a general analysis. The colonoscopy was performed after standard bowel preparation and sedation (midazolam and meperidine). The total procedure was not traumatic, and no excess air insufflation was used. There was a small polyp in the left colon. However, on entering the cecal area, several bright linear marks were seen in the mucosa (Figures 1 and 2) with some extravasation of fresh blood. A couple of biopsies were taken which were informed as normal. The polyp resected on withdrawal was adenomatous. The remainder of the colorectal mucosa was macroscopically normal.

“Cat scratch colon” is defined as bright erythematous linear marks, sometimes with extravasation of little fresh blood, seen in the cecum and in ascending colon. It is a rare colonoscopy finding first described by McDonnell et al. in 2006 [1]. The authors reviewed 8277 colonoscopies performed in a single endoscopy center and identified 21 cases, mainly female patients. All these patients were biopsied and normal findings were seen in histological specimens except in 2 of them, where collagenous colitis was described. 

The etiology is not well known. Vascular malformations do not play a role, and inflammation is not seen in the biopsies taken. The lesions are not due to direct scope trauma. We detected them, as other authors did, previous to the intubation of the area affected. McDonnell et al. suggested these scratches can represent barotraumas from air insufflation into a less compliant colon during the colonoscopy. Distension and traction during a special difficult procedure can be the cause, as hemorrhagic colonic mucosa can occasionally be seen in such cases [2]. Tominaga et al. [3] published two nice pictures of a female patient: a first gentle intubation of the ascending colon was performed, where colonic mucosa was normal. A second, more difficult one—in the same procedure—was done in which these mucosal superficial breaks appeared. This supports the evidence of a barotrauma etiology. 

There is also an interesting paper published by Cruz-Correa et al. [4], where they describe these lacerations in 3 patients affected of collagenous colitis. Their etiological hypothesis is a combination of a less compliant colon due to the thick collagen submucosa layer and endoscopic insufflation. However, Yarze [5] argues that collagen does not play such a main role. In his opinion, the lacerations could just be explained with Laplace's law (“the tension in the wall of a cylindrical vessel is proportional to the ratio”). The right colon and the cecum have the greatest diameter. 

Some other authors such as Baudet et al. [6] observed this type of lesion related to diversion colitis. They found lacerations on withdrawing the air from a rectal stump. In their opinion, these findings confirm the barotrauma etiology in an otherwise altered, less compliant colonic mucosa. This hypothesis is similar to Cruz-Correa's.

However, although this colonic sign is consider benign, Purnak et al. [7] have recently described a 50-year-old patient with chronic cholestasis due to cholangiocellular carcinoma with these special linear marks. They hypothesized an epithelial disruption and tendency to bleed due to vitamin A and K deficiency and impair gut's barrier in the huge intestinal oxidative stress which occurs in obstructive jaundice. Katsinelos et al. [8] observed a 73-year-old man suffering from metastatic liver disease with these bright linear marks on the ascending colon. They suggest that barotrauma together with a less compliant right colon due to the pressure from the liver could have played a role. There are also some other hypothesis such as chronic anti-inflammatory drug ingestion as recently reported in a Spanish publication [9].

We agree with previous authors that the lesions in our patient can be due to barotraumas. Furthermore, heavy smoking and microvascular disease in this patient added to the ingestion of acetyl salicylic acid, which could have played a role to their development.

## Figures and Tables

**Figure 1:**
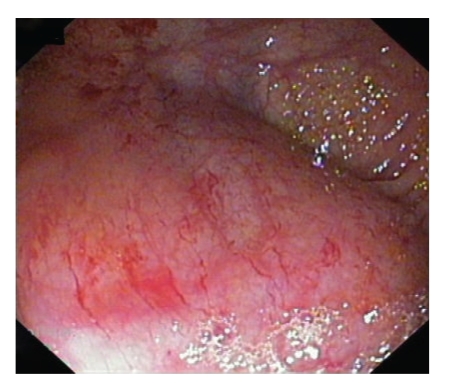


**Figure 2:**